# EGFR TKIs impair lysosome-dependent degradation of SQSTM1 to compromise the effectiveness in lung cancer

**DOI:** 10.1038/s41392-019-0059-4

**Published:** 2019-07-12

**Authors:** Lixian Yang, Shilong Ying, Shiman Hu, Xiangtong Zhao, Muchun Li, Miaoqin Chen, Yiran Zhu, Ping Song, Liyuan Zhu, Tingting Jiang, Huimin An, Neelum Aziz Yousafzai, Wenxia Xu, Zhiguo Zhang, Xian Wang, Lifeng Feng, Hongchuan Jin

**Affiliations:** 1 Laboratory of Cancer Biology, Key Lab of Biotherapy in Zhejiang, Sir Run Run Shaw Hospital, Medical School of Zhejiang University, Hangzhou, China; 2 Department of Medical Oncology, Key Lab of Biotherapy in Zhejiang, Sir Run Run Shaw Hospital, Medical School of Zhejiang University, Hangzhou, China; 3 Department of Pathology, Sir Run Run Shaw Hospital, Medical School of Zhejiang University, Hangzhou, China; 4 Key Laboratory of Biomass Chemical Engineering of Ministry of Education, College of Chemical and Biological Engineering, Zhejiang University, Hangzhou, China

**Keywords:** Lung cancer, Cancer therapy, Cancer therapy

## Abstract

Tyrosine kinase inhibitors for epidermal growth factor receptor (EGFR TKIs) greatly improved clinical outcomes of patients with non-small cell lung cancer (NSCLC). Unfortunately, primary and acquired resistance limits their clinical benefits. To overcome such resistance, new generations of EGFR TKIs have been developed by targeting newly identified mutations in *EGFR*. However, much less effort has been put into alternative strategies, such as targeting the intrinsic protective responses to EGFR TKIs. In this study, we found that EGFR TKIs, including gefitinib and AZD9291, impaired lysosome-dependent degradation of SQSTM1, thus compromising their anti-cancer efficiency. By accumulating in the lysosome lumen, gefitinib and AZD9291 attenuated lysosomal acidification and impaired autolysosomal degradation of SQSTM1 owing to their intrinsic alkalinity. As a result, SQSTM1 protein was stabilized in response to gefitinib and AZD9291 treatment and conferred EGFR TKI resistance. Depleting SQSTM1 significantly increased the sensitivity of NSCLC cells to gefitinib and AZD9291 both in vitro and in vivo. Furthermore, a chemically modified gefitinib analog lacking alkalinity displayed stronger inhibitory effects on NSCLC cells. Therefore, targeting accumulated SQSTM1 or chemically modified EGFR TKIs may represent new strategies to increase the effectiveness of EGFR targeted therapy.

## Introduction

Lung cancer is the most frequent cause of cancer-associated death worldwide, and NSCLC (non-small cell lung cancer) accounts for 80% of lung cancers.^1^ Although NSCLC patients initially respond to chemotherapy, the duration of the response is often limited, and NSCLC patients with multiple relapses are generally resistant to chemotherapy. Fortunately, small molecule TKIs (tyrosine kinase inhibitors) of EGFR (epidermal growth factor receptor) have been accepted as the first-line treatment option for NSCLC patients with mutant *EGFR.*^2^ Unfortunately, almost all NSCLC patients who originally respond to EGFR TKI treatment eventually experience disease progression due to acquired resistance, resulting in a limited 5-year survival rate of NSCLC patients that ranges from 4 to 17%.^3^ Resistance to EGFR TKIs develops predominantly through the following pathways: secondary mutations of EGFR gene, compensatory activation of other growth factor receptors and activation of signaling molecules downstream of EGFR.^4^ The T790M (Thr790Met) mutation is the most common secondary *EGFR* mutation conferring resistance to first-generation EGFR TKIs, leading to the development of a third-generation TKI, AZD9291 (osimertinib).^5^ However, acquired resistance to this new-generation EGFR TKI is developing relentlessly.^6^ Therefore, alternative strategies are urgently needed to overcome TKI resistance.

Macroautophagy (hereafter referred to as autophagy) is a process delivering protein aggregates and damaged organelles that are enclosed in double-membrane vesicles to lysosomes for degradation.^7^ Generally, the process of autophagy is divided into four consecutive steps: autophagy initiation, autophagosome formation, autolysosomal fusion, and autolysosomal degradation.^8^ Accumulating evidence suggests that autophagy has a fundamental effect on the clinical outcome of chemotherapy and targeted therapy. The majority of chemotherapy agents and targeted therapeutics have been found to induce protective autophagy, resulting in reduced sensitivity to antitumor drugs, including traditional chemotherapy drugs (e.g., platinum compounds,^9^ fluorouracil,^10^ and etoposide^11^) and molecular targeted agents (e.g., bevacizumab,^12^ cetuximab,^13^ and EGFR TKIs^14,15^) As a consequence, autophagy inhibitors such as CQ (chloroquine) and HCQ (hydroxychloroquine) have been extensively investigated to potentiate the sensitivity to antitumor drugs.^16^ However, several phase I and II clinical trials showed only a moderate improvement in the antitumor activity of several chemotherapeutics.^17^ The underlying mechanisms remain largely elusive.

As an adaptor protein that delivers cargos into double-membrane vesicles for autophagic degradation, SQSTM1 (sequestosome 1)/p62 plays a critical role in autophagy.^18^ Interestingly, SQSTM1 also contributes to tumorigenesis and drug resistance by activating multiple oncogenic signaling pathways, such as Nrf2 (nuclear factor erythroid 2-related factor 2, NFE2L2)-ROS (reactive oxygen species),^19^ NFκB (nuclear factor kappa-light-chain-enhancer of activated B cells),^20^ and mTORC1 (mammalian target of rapamycin complex 1) pathways.^21^ Since SQSTM1 is also a substrate of autophagic degradation, the inactivation of autophagy eventually leads to its accumulation. Therefore, SQSTM1 accumulation may compromise the effect of autophagy inhibitors such as CQ and HCQ.

In this study, we found that EGFR TKIs upregulated SQSTM1 expression in NSCLC cells by repressing its autolysosomal degradation. Knockdown of SQSTM1 expression significantly sensitized NSCLC cells to EGFR TKIs both in vitro and in vivo. Mechanistically, EGFR TKIs attenuated lysosomal acidification owing to their intrinsic alkalinity, and the modification of gefitinib to diminish its alkalinity led to an enhanced inhibitory effect on NSCLC compared with parental gefitinib. Therefore, targeting SQSTM1 accumulation or chemically modifying EGFR TKIs might be valuable strategies for overcoming EGFR TKI resistance.

## Materials and methods

### Cell lines, antibodies, and reagents

A549, HCC827, and H460 cells were purchased from the Cell Bank of the Chinese Academy of Science (Shanghai, China). HCC827-R cells were kindly supplied by Dr. Jiawei Shou.^22^ Anti-SQSTM1 antibodies (rabbit, pm045; mouse, m162-3) were obtained from Medical & Biological Laboratories (Nagoya, Japan). The anti-LAMP1 (ab25630) and anti-p-Akt (S473) antibodies (ab81283) were obtained from Abcam (Shanghai, China). The anti-β-actin (4970), anti-LC3 (2775), anti-cleaved PARP1 (9541), anti-p-P70S6K (T389) (9205), and anti-cathepsin B antibodies (31718) were obtained from Cell Signaling Technology (Shanghai, China). The anti-Akt antibody (1085-1) was purchased from Epitomics (Hangzhou, China). Gefitinib (S1025), AZD9291 (S7292), and bafilomycin A1 (S1413) were purchased from Selleck (Shanghai, China). Cycloheximide (R750107), chloroquine (C6628), and acridine orange (A8097) were purchased from Sigma Aldrich (Shanghai, China). Rapamycin (S1842) was purchased from Beyotime (Shanghai, China). LysoTracker® Blue DND-22 (L7525) was purchased from Invitrogen (Shanghai, China).

### SiRNA and plasmid transfections

For siRNA transfections, cells were seeded overnight and transfected with Lipofectamine^TM^ RNAiMAX transfection reagent (Thermo Fisher Scientific, Shanghai, China) according to the manufacturer’s protocol. After 48 h, EGFR TKIs were added for another 24 or 48 h. For plasmid transfections, cells were seeded overnight, and plasmids were transfected with X-treme GENE HP DNA Transfection Reagent (Roche Applied Science, Shanghai, China) according to the manufacturer’s instructions.

### Cell viability and apoptosis assay

Cells were seeded into 96-well plates and treated as indicated. The CellTiter 96 AQueous Non-Radioactive Cell Proliferation Assay kit (Promega, Beijing, China) was used for cell viability analysis. Both western blotting and flow cytometry analysis were used for the detection of apoptosis. For flow cytometry analysis, the cells were collected by trypsinization and centrifugation at 1000 × *g* for 5 min and stained with the BD Pharmingen FITC Annexin V Apoptosis Detection Kit (BD Biosciences, Shanghai, China). The samples were analyzed with a BD FACSCalibur^TM^ flow cytometer (BD Biosciences) within 1 h.

### Western blotting

Cells were lysed with lysis buffer containing 2% SDS, 0.1% bromophenol blue, 10% glycerinum, 1.5% DTT (dithiothreitol), and 0.1 M Tris-HCl (pH 6.8). Cell lysates were quantitated by a BCA protein assay kit (Bio-Rad Laboratories, Hercules, CA, USA). The boiled lysates were separated by SDS-PAGE, transferred to polyvinylidene fluoride membranes and blotted with 5% non-fat milk (dissolved in TBST). Then, the membranes were incubated with the indicated primary antibodies at 4 °C overnight and then with the horseradish peroxidase-conjugated secondary antibody (Jackson ImmunoResearch, Shanghai, China). TBST (TBS with 0.1% Tween-20) was used to wash the membranes following antibody incubation. Ultimately, the membranes were visualized in an Amersham Imager 600 system (GE Healthcare Life Science, Shanghai, China) with a Chemiluminescence Detection Kit for HRP (Biological Industries, Cromwell, USA).

### RNA extraction and quantitative real-time RT-PCR

Total RNA was extracted with TRIzol reagent (Invitrogen, Shanghai, China) according to the manufacturer’s instructions, and RNA concentration was quantified by NanoDrop 2000 (Nanodrop, Wilmington, DE, USA). RNA (1–2 μg) was reverse transcribed with the High Capacity cDNA Reverse Transcription Kit (Thermo Fisher Scientific). Relative mRNA levels were detected by quantitative real-time PCR with SYBR Green Master Mix Kit (ComWin Biotech, Beijing, China) and a Light Cycler 480 II system (Roche Applied Science). The SQSTM1 primer sequences were previously reported.^23^

### Immunofluorescence and confocal microscopy

Cells were seeded on coverslips, incubated overnight and treated as indicated. Briefly, the cells were fixed with cold methanol for 10 min, permeabilized in 0.2% Triton X-100 for 12 min and blotted with 3% BSA (bovine serum albumin; diluted in PBS) for 30 min. The appropriate primary antibodies were diluted with 3% BSA and incubated with the cells at 4 °C overnight. Then, the cells were washed three times with 0.5% PBS-T (PBS with 0.5% Tween-20), incubated with the appropriate secondary antibodies for 1 h at room temperature, and sealed with mounting medium including DAPI after being washed. Images were captured on a Nikon A1 laser scanning confocal microscope (Nikon, Shanghai, China).

### Lysosomal tracking and AO staining

Cells were seeded on coverslips and incubated with 0.5 μM LysoTracker Blue for 2 h or with 5 μg/ml AO for 15 min at 37 °C. After being washed with PBS three times, the cells were sealed with 20% glycerinum (diluted in PBS) and then visualized immediately with an Olympus laser scanning microscope.

### Tandem mRFP-GFP fluorescence microscopy

To detect tandem fluorescent LC3 puncta, the cells were transfected for 48 h with the tfLC3 plasmid. The transfected cells were sealed with 20% glycerinum and then visualized by a Nikon A1 laser scanning confocal microscope.

### Immunohistochemistry

Formalin-fixed and paraffin-embedded cancer tissue sections were immunostained with anti-SQSTM1 or anti-cleaved-caspase 3 antibodies after microwave antigen retrieval in 0.01 M citrate buffer (pH 6.0). After a washing step, the signal was detected using a suitable HRP-labeled secondary antibody with DAB as the chromogen (Dako, Denmark).

### Chemical synthesis of fluorescein-labeled gefitinib (Gefi-RB) and gefitinib-2OH (Gefi-2OH)

Fluorescein-labeled gefitinib (Gefi-RB) was synthesized through a nucleophilic substitution reaction of demethylated gefitinib with rhodamine B alkyl bromide analog **3** (Supplemental Fig. 6A). Briefly, these two intermediate compounds were synthesized according to reported previously methods with modifications.^24,25^

The synthesis of gefitinib-2OH (Gefi-2OH) started with preparing the corresponding THP-protected benzenediol (Supplemental Fig. 7A). Accordingly, 3,5-bis((tetrahydro-2H-pyran-2-yl)oxy)benzoate was synthesized through the acylation reaction, AlCl_3_-catalyzed demethylation, and phenolic hydroxyl THP protection. This protected intermediate was then reacted with demethylated gefitinib and deprotected by 10% HCl solution to produce Gefi-2OH.

The detailed synthesis procedures for Gefi-RB and Gefi-2OH are provided in the supplementary section. The structure of each intermediate was confirmed by ^1^H-NMR, and the structure of the target product was analyzed by ^1^H-NMR and mass spectrometry.

### pKa value calculations

Computer programs for pKa estimations of EGFR TKIs and Gefi-2OH were applied in the commonly used package Marvin from ChemAxon.^24,25^ The structures of gefitinib and Gefi-2OH were drawn by MarvinSketch. The general calculation mode was set as follows: the macro mode was used to estimate the macro acidic dissociation constant; the static acid/base prefix was chosen to estimate the pKa value of neutral acidic and basic sites by red and blue annotation, and the pKa range was set according to the defaults (min basic pKa = −2, max acidic pKa = 16).

### In vivo tumorigenicity assay

Female nude mice (6–8 weeks old) were purchased from Shanghai Laboratory Animal Center, CAS (SLACCAS) and maintained under specific pathogen-free conditions. The mice were then randomly divided into two groups and subcutaneously injected with 5 × 10^6^ A549 or A549-SQSTM1 cells. Three days after inoculation, gefitinib was administered every 2 days by oral gavage at 75 mg/kg in 1% Tween 80 (Sigma) in sterile Milli-Q water (the vehicle control was 1% Tween 80 in sterile Milli-Q water). SQSTM1 shRNA sequence (same sequence as the siRNA anti-SQSTM1-1#) and a scramble shRNA were packaged into recombinant lentivirus by Genechem Company (Shanghai, China). The lentiviruses containing SQSTM1 shRNA and scramble shRNA were named shSQSTM1 and shNC, respectively. Then, 1 × 10^7^ A549 cells were subcutaneously inoculated into the nude mice. Once the tumors were palpable, the mice were randomly divided into two groups and received four intratumoral injections of shSQSTM1 or shNC (50 μl, 1 × 10^8^ TU/ml). Three days after the lentivirus injections, gefitinib was administered as described above.

Tumor volume was measured with Vernier calipers every 2 days after the start of gefitinib administration. Tumors were harvested and weighed 28 days after inoculation. Student’s *t* test was performed for statistical analysis, and a *p* value < 0.05 was considered statistically significant.

## Results

### EGFR TKIs upregulate SQSTM1 expression in NSCLC cell lines

**Fig. 1:**
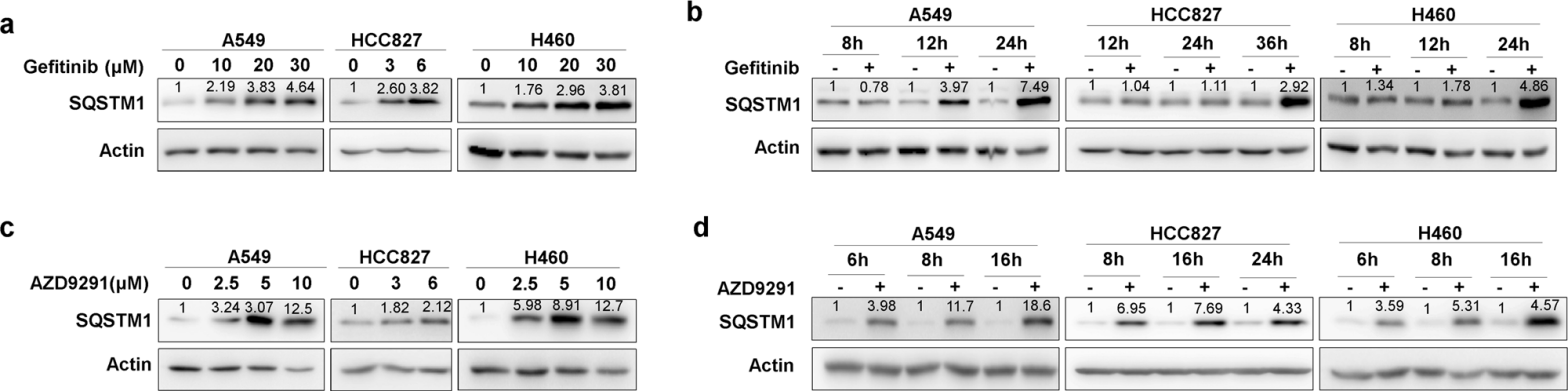
Gefitinib and AZD9291 upregulate SQSTM1 in NSCLC. **a** Non-small lung cancer cells (A549, HCC827, and H460) were treated with gefitinib for 24 h (A549 and H460) or 36 h (HCC827) at the indicated concentration, and the expression of SQSTM1 was measured by western blotting. Actin was used as the loading control. **b** A549, HCC827, and H460 cells were treated with gefitinib at 20 μM (A549 and H460) or 6 μM (HCC827) for the indicated time, and the expression of SQSTM1 was measured by western blotting. **c** A549, HCC827, and H460 cells were treated with AZD9291 for 24 h at the indicated concentration, and the expression of SQSTM1 was measured by western blotting. **d** A549, HCC827, and H460 cells were treated with AZD9291 at 10 μM (A549 and H460) or 6 μM (HCC827) for the indicated time, and the expression of SQSTM1 was measured by western blotting. Relative SQSTM1 expression (SQSTM1/Actin) was quantified and normalized to the relative SQSTM1 expression in the control

We started to explore the effect of various EGFR TKIs on the expression of SQSTM1. Early generation EGFR TKIs, such as gefitinib and erlotinib, upregulated the protein level of SQSTM1 in a dose- and time-dependent manner in three NSCLC cells with different genetic backgrounds, A549 (wild-type *EGFR*), HCC827 (exon 19 deletion in *EGFR*), and NCI-H460 cells (H460, wild-type *EGFR*) (Fig. 1a, b, Supplemental Fig. 1). Interestingly, the new-generation EGFR TKI AZD9291 also upregulated SQSTM1 protein in a dose- and time-dependent manner in all three NSCLC cell lines (Fig. 1c, d).

### SQSTM1 knockdown sensitizes NSCLC cells to gefitinib and AZD9291

**Fig. 2:**
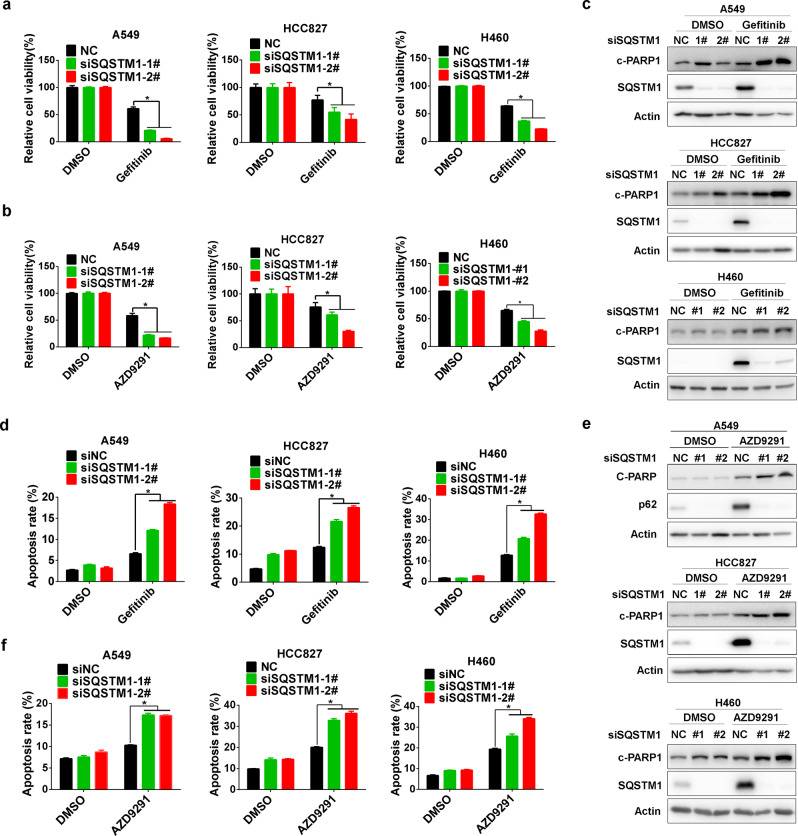
SQSTM1 knockdown sensitized NSCLC to gefitinib and AZD9291. Gefitinib (**a**) or AZD9291 (**b**) was administered to A549, HCC827, and H460 cells for 48 h after transfection with control siRNA (siNC) or SQSTM1 siRNA (siSQSTM1). Cell viability was measured by MTS assay. **c** A549, HCC827, and H460 cells were treated with gefitinib after 48 h of siSQSTM1 transfection. Western blotting was applied to measure c-PARP1 (cleaved PARP1) levels. **d** A549, HCC827, and H460 cells were treated with gefitinib after 48 h of siSQSTM1 transfection. The annexin V-PI staining assay was performed after the indicated treatment, and the relative percentage of apoptotic cells is summarized. **e** A549, HCC827, and H460 cells were treated with AZD9291 after 48 h of siSQSTM1 transfection. Western blotting was applied to measure c-PARP1 levels. **f** A549, HCC827, and H460 cells were treated with AZD9291 after 48 h of siSQSTM1 transfection. The annexin V-PI staining assay was performed after the indicated treatment, and the relative percentage of apoptotic cells is summarized. All statistical data are presented as the mean ± SD. Student’s *t* test was used for the statistical analysis, and a *p* value < 0.05 was considered statistically significant

Next, we analyzed the relevance of SQSTM1 upregulation to EGFR TKI sensitivity. In the absence of EGFR TKIs, SQSTM1 knockdown had a moderate effect on the viability of NSCLC cells (Supplementary Fig. 2A, B). However, it significantly potentiated EGFR TKI-induced inhibition of viability in all three NSCLC cell lines (Fig. 2a, b), which was accompanied by increased activation of apoptosis (Fig. 2c–f; Supplementary Fig. 2C, D).

**Fig. 3:**
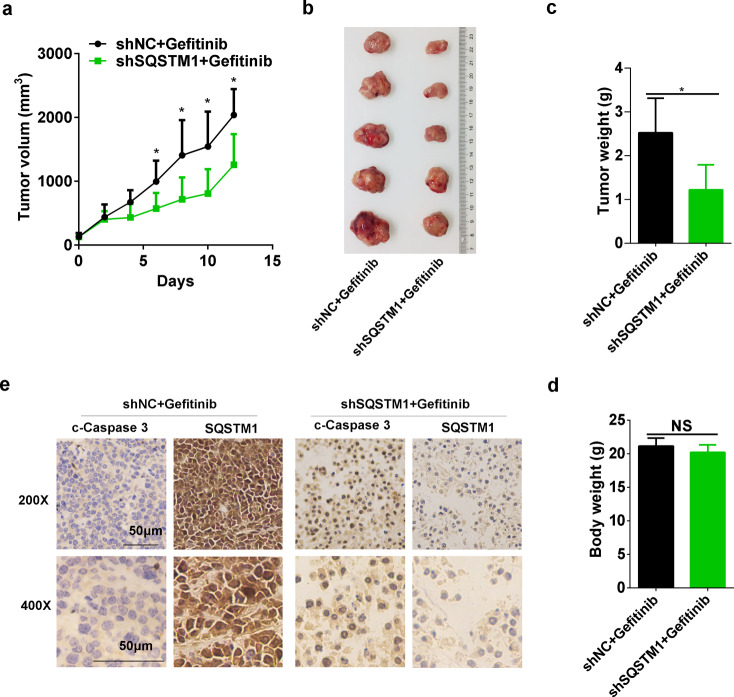
SQSTM1 knockdown increased the sensitivity to gefitinib by inducing apoptosis in vivo. The in vivo effect of SQSTM1 knockdown on the sensitivity to gefitinib was measured in A549 xenograft tumors that received an intratumoral injection of shNC (*n* = 5) or shSQSTM1 lentivirus (*n* = 5). The tumor volume curve is shown in (**a**). Photographs and weights of the final harvested tumors are shown in (**b**) and (**c**). **d** Body weight in the two groups of mice. NS, not significant (Student’s *t* test, *p* > 0.05). **e** Immunohistochemical staining of paraffin-embedded tissue sections for SQSTM1 and c-caspase 3 (cleaved caspase 3). Scale bar, 50 μm. Data are presented as the mean ± SD. Student’s *t* test was used for the statistical analysis, and a *p* value < 0.05 was considered statistically significant

Consistently, the in vivo growth of NSCLC cells treated with gefitinib was significantly impeded by SQSTM1 knockdown (Fig. 3a). After gefitinib treatment, the tumors formed by NSCLC cells with SQSTM1 knockdown were much smaller than those formed by NSCLC cells without SQSTM1 knockdown (Fig. 3b, c). While the mouse body weights were similar (Fig. 3d), more apoptosis was observed in NSCLC cells with SQSTM1 knockdown (Fig. 3e). In summary, both the in vitro and in vivo results demonstrated the potentiation of gefitinib and AZD9291 efficacy by reduced SQSTM1 expression.

### SQSTM1 attenuates growth inhibition by gefitinib and AZD9291 by repressing apoptosis

**Fig. 4:**
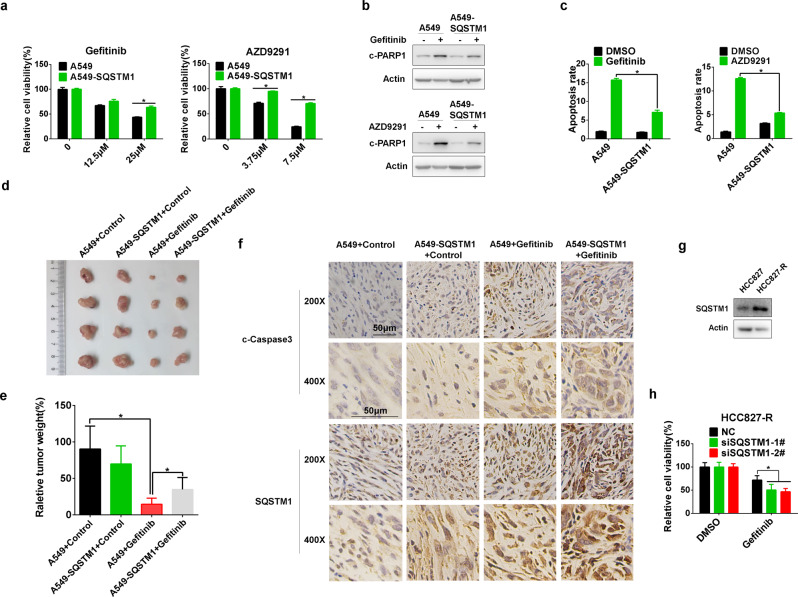
SQSTM1 overexpression confers gefitinib and AZD9291 resistance in vitro and in vivo. **a** A549 and A549-SQSTM1 cells were treated with gefitinib or AZD9291 at the indicated concentration for 48 h, and an MTS assay was performed to measure cell viability. **b** A549 and A549-SQSTM1 cells were treated with gefitinib or AZD9291 for 48 h, and western blotting was applied to measure c-PARP1 levels. **c** The annexin V-PI staining assay was performed after the indicated treatments, and the relative percentage of apoptotic cells is summarized. The in vivo effect of SQSTM1 overexpression on sensitivity to gefitinib was measured in an A549 or A549-SQSTM1 xenograft tumor growth assay. Photographs and weights of the final harvested tumors are shown in (**d**) and (**e**). The tumor weight in each group is shown as the mean ± SD. Asterisks indicate significant differences (Student’s *t* test, *p* < 0.05). **f** Immunohistochemical staining of paraffin-embedded tissue sections for SQSTM1 and c-caspase 3. Scale bar, 50 μm. **g** Western blotting was used to detect the expression level of SQSTM1 in HCC827 and HCC827-R cells. **h** HCC827-R cells were treated with gefitinib after transfection with siNC or siSQSTM1 for 48 h, and cell viability was detected by MTS assay

Meanwhile, A549 cells overexpressing SQSTM1 (A549-SQSTM1) (Supplemental Fig. 3A) displayed resistance to gefitinib and AZD9291 compared with parental A549 cells (Fig. 4a). EGFR TKI-induced apoptosis was significantly reduced in A549-SQSTM1 cells (Fig. 4b, c; Supplementary Fig. 3B). In addition, gefitinib significantly inhibited the in vivo growth of A549 cells in nude mice (Fig. 4d, e). However, this inhibitory effect was greatly hampered in A549-SQSTM1 cells (Fig. 4d, e). Meanwhile, much less apoptosis was observed in gefitinib-treated A549-SQSTM1 cells (Fig. 4f). Interestingly, in a gefitinib-resistant NSCLC cell (HCC827-R)^22^ (Supplementary Fig. 3C), SQSTM1 expression was evidently increased (Fig. 4g). Moreover, gefitinib treatment dramatically elevated SQSTM1 expression in these cells (Supplementary Fig. 3D), and knockdown of its expression significantly reversed gefitinib resistance and augmented gefitinib-induced apoptosis (Fig. 4h; Supplementary Fig. 3E, F). Collectively, these results indicate that enhanced expression of SQSTM1 contributes to gefitinib and AZD9291 resistance in NSCLC cells by repressing apoptosis.

### Gefitinib and AZD9291 induce SQSTM1 accumulation by inhibiting its autophagic degradation

**Fig. 5:**
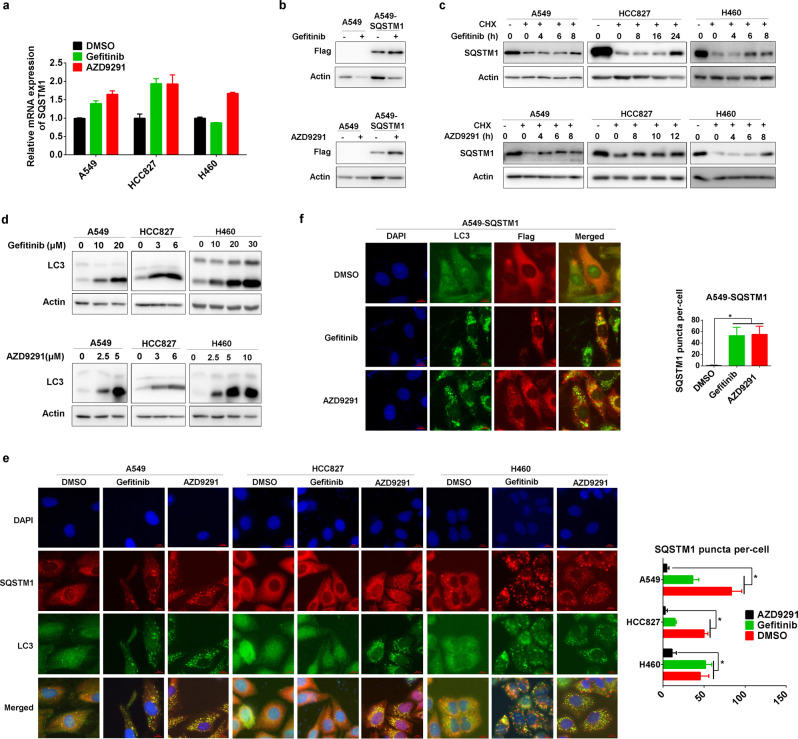
Gefitinib and AZD9291 repress the autophagic degradation of SQSTM1. **a** A549, HCC827, and H460 cells were treated with gefitinib or AZD9291, and SQSTM1 mRNA expression was assessed by qRT-PCR. The experiments were performed in triplicate, and representative results are shown as the mean ± SD. **b** A549 and A549-SQSTM1 cells were treated with gefitinib (upper panel) or AZD9291 (lower panel) for 24 h, and the expression of Flag-SQSTM1 was measured by western blotting. **c** A549, HCC827, and H460 cells were treated with CHX for 8, 12, or 24 h and cotreated with gefitinib or AZD9291 for the indicated times. The expression of SQSTM1 was detected by western blotting. **d** A549, HCC827, and H460 cells were treated with gefitinib or AZD9291 for 24 h, and the expression of LC3-I and LC3-II was measured by western blotting. **e** A549, HCC827, and H460 cells were treated with gefitinib or AZD9291 for 24 h, and SQSTM1 and LC3 puncta were detected by immunofluorescence microscopy. Scale bar: 10 μm. The quantification of SQSTM1 puncta per cell is expressed as the mean ± SD (*n* = 10 cells, three independent experiments). Asterisks indicate significant differences (Student’s *t* test, *p* < 0.05). **f** A549-SQSTM1 cells were treated with gefitinib or AZD9291 for 24 h, and FLAG-SQSTM1 and LC3 puncta were detected by immunofluorescence microscopy. Scale bar: 10 μm. The quantification of FLAG-SQSTM1 puncta per cell is expressed as the mean ± SD (*n* = 10 cells, three independent experiments). Asterisks indicate significant differences (Student’s *t* test, *p* < 0.05)

Next, we wondered how gefitinib and AZD9291 increase SQSTM1 expression in NSCLC cells. Gefitinib and AZD9291 only slightly upregulated SQSTM1 mRNA levels (Fig. 5a). However, gefitinib and AZD9291 significantly increased the protein level of exogenous SQSTM1 (Fig. 5b). Importantly, gefitinib and AZD9291 were able to increase the protein level of SQSTM1 even in the presence of the translation inhibitor CHX (cycloheximide) (Fig. 5c). Taken together, the data suggest that gefitinib and AZD9291 most likely upregulate SQSTM1 expression by repressing the degradation of SQSTM1 protein rather than stimulating its protein synthesis or gene transcription.

In addition to delivering polyubiquitinated proteins for autophagic degradation, SQSTM1 itself is degraded by autophagy.^18^ The blockade of autophagy eventually leads to the accumulation of SQSTM1. Indeed, gefitinib and AZD9291 simultaneously increased SQSTM1 accumulation and LC3I to LC3II conversion (Figs. 1a–d and 5d; Supplementary Fig. 1), which was similar to the effects of the well-known autophagy inhibitor CQ.^26^ In addition, gefitinib and AZD9291 increased the aggregation of endogenous and exogenous SQSTM1 colocalizing with LC3 in the cytoplasm (Fig. 5e, f). Taken together, these results indicate that gefitinib and AZD9291 inactivate autophagy, leading to the accumulation of SQSTM1.

### Gefitinib and AZD9291 block autophagy flux

**Fig. 6:**
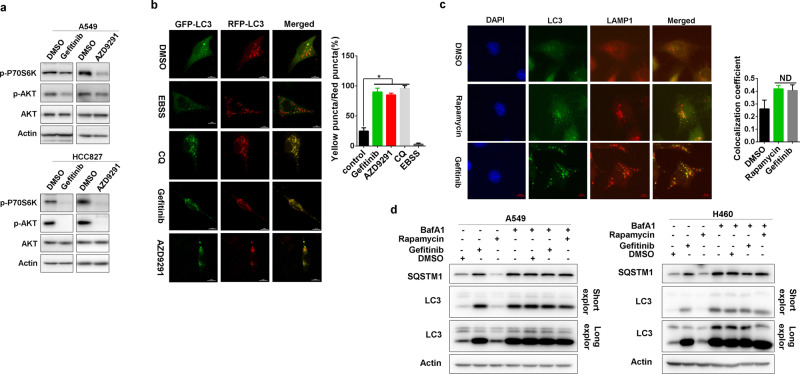
Gefitinib and AZD9291 repress autophagic degradation. **a** A549 and HCC827 cells were treated with gefitinib or AZD9291 for 12 h. The phosphorylation of mTOR (p-mTOR) and Akt (p-Akt) was analyzed by western blotting. **b** A549 cells were transfected with the GFP-RFP-LC3 plasmid for 48 h and then treated with EBSS, CQ, gefitinib, or AZD9291. Immunofluorescence microscopy was performed to detect GFP-LC3 and RFP-LC3 puncta. The quantification of LC3 yellow puncta/red puncta (%) is expressed as the mean ± SD (*n* = 10 cells, three independent experiments). Asterisks indicate significant differences (Student’s *t* test, *p* < 0.05). **c** A549 cells were treated with DMSO, rapamycin or gefitinib for 24 h, and immunofluorescence was performed to detect the colocalization of LAMP1 and LC3. The colocalization coefficient is presented as the ratio of colocalizing LC3 and LAMP1 puncta versus total LC3 puncta. The quantification of the colocalization coefficient is expressed as the mean ± SD (*n* = 10 cells, three independent experiments). NS, not significant (Student’s *t* test, *p* > 0.05). **d** A549 and H460 cells were pretreated with DMSO, rapamycin, or gefitinib for 7 h and then treated with or without BafA1 for 5 h. LC3 and SQSTM1 expression was detected by western blotting

However, gefitinib and AZD9291 effectively suppressed PI3K/mTOR signaling (Fig. 6a), which should result in the initiation of autophagy. Indeed, we did find increased LC3 puncta upon gefitinib and AZD9291 treatment (Supplementary Fig. 4). We therefore analyzed autophagy flux after the treatment with gefitinib and AZD9291 using a tandem mRFP-GFP-tagged LC3 (tfLC3) reporter.^26^ The green (GFP) signal is sensitive to the acidic condition in the lysosomal lumen, the yellow signal resulting from the colocalization of GFP and mRFP fluorescence represents autophagosomes before fusion with acidic lysosomes, and the red (mRFP) signal indicates acidic autolysosomes.^27^ In contrast to the well-known autophagy inducer EBSS (Earle’s balanced salt solution), gefitinib and AZD9291 increased autophagosomes only, not autolysosomes (Fig. 6b). This result was quite similar to that of the classical autophagy flux blocker CQ (Fig. 6b), indicating that gefitinib and AZD9291 blocked, rather than activated, autophagy flux at the late stage of autophagy.

This blockade could result from defects in the formation of autolysosomes or the function of lysosomes. Gefitinib induced the colocalization of LC3 and LAMP1 (lysosomal-associated membrane protein 1) nearly as well as rapamycin (Fig. 6c), indicating that gefitinib and AZD9291 might not affect the formation of autolysosomes. However, in contrast to rapamycin, which increased both SQSTM1 accumulation and LC3 I to LC3 II conversion only in the presence of BafA1 (Bafilomycin A1, another autophagy inhibitor^28^) gefitinib-induced SQSTM1 accumulation and LC3 I to LC3 II conversion irrespective of BafA1 presence (Fig. 6d). Therefore, gefitinib and AZD9291 blocked the autophagic degradation of SQSTM1, most likely by repressing the function of lysosomes.

### Gefitinib and AZD9291 repress lysosomal acidification independent of EGFR signaling

**Fig. 7:**
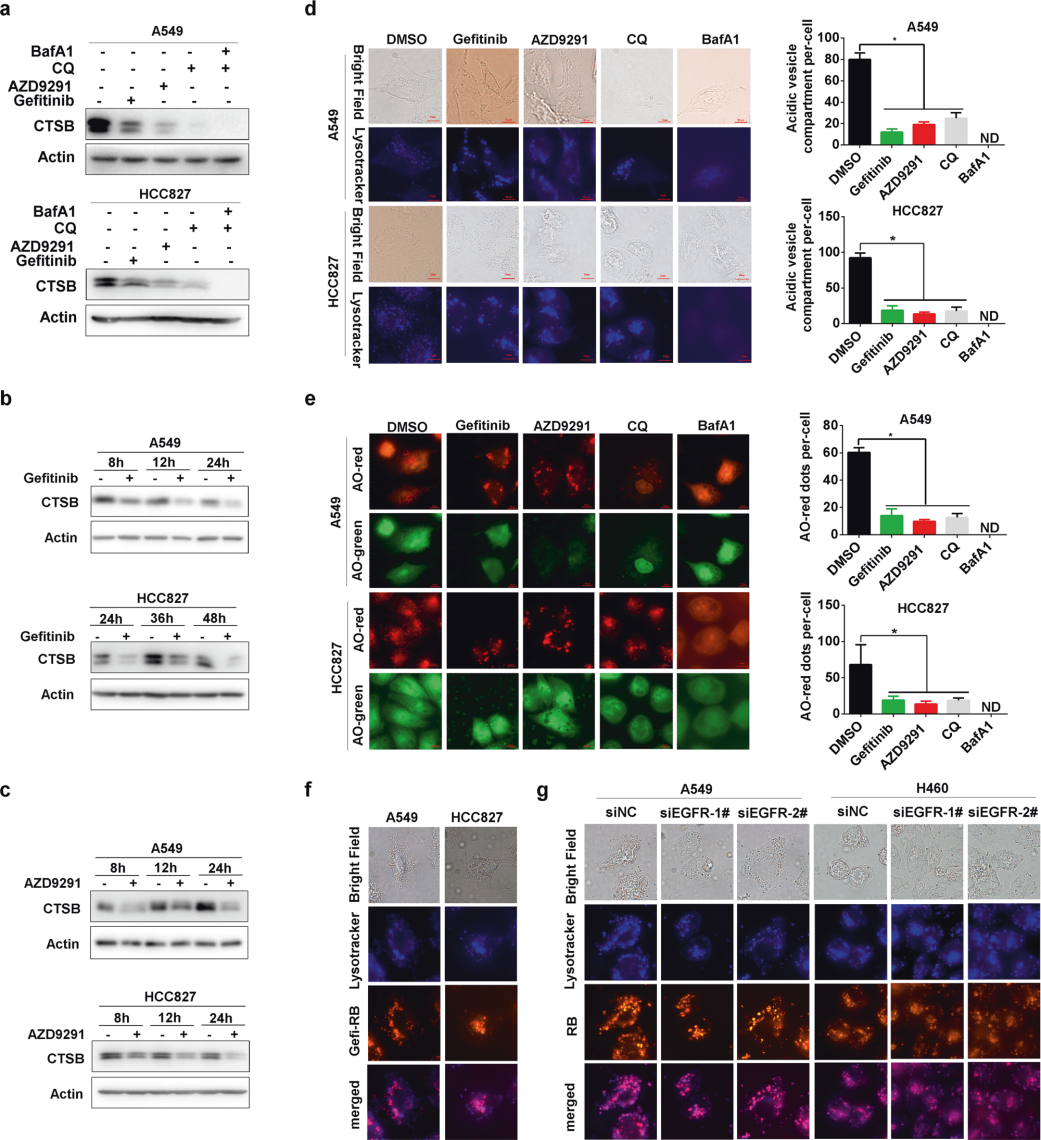
Gefitinib and AZD9291 repress lysosomal acidification independent of EGFR signaling. **a** A549 and HCC827 cells were treated with gefitinib, AZD9291, CQ, or BafA1 for 24 h, and the expression of mature CTSB was assessed by western blotting. A549 and HCC827 cells were treated with gefitinib (**b**) or (**c**) AZD9291 for the indicated times, and the expression of mature CTSB was assessed by western blotting. **d** A549 and HCC827 cells were treated with DMSO, gefitinib, AZD9291, CQ, or BafA1 for 12 h and then incubated with LysoTracker Blue for 2 h. Fluorescent microscopy was used to detect the number of acidic vesicles. Scale bar: 10 μm. The quantification of acidic vesicle compartments per cell is expressed as the mean ± SD (*n* = 10 cells, three independent experiments). Asterisks indicate significant differences (Student’s *t* test, *p* < 0.05), and ND represents not detected. **e** A549 and HCC827 cells were treated with DMSO, gefitinib, AZD9291, CQ, or BafA1 for 12 h and then incubated with AO for 15 min. Fluorescence microscopy was used to detect the number of AO-red dots. Scale bar: 10 μm. The quantification of AO-red dots per cell is expressed as the mean ± SD (*n* = 10 cells, three independent experiments). Asterisks indicate significant differences (Student’s *t* test, *p* < 0.05). ND, not detected. **f** A549 and HCC827 cells were treated with gefitinib or Gefi-RB (gefitinib labeled with rhodamine B) and then incubated with LysoTracker Blue. Fluorescence microscopy was used to detect the colocalization of gefitinib and acidic vesicles. Scale bar: 10 μm. **g** A549 and HCC827 cells were treated with Gefi-RB after knocking down EGFR for 24 h and then incubated with LysoTracker Blue. Fluorescence microscopy was used to detect the colocalization of gefitinib and acidic vesicles. Scale bar: 10 μm

To prove it, we explored the effect of gefitinib and AZD9291 on the level of mature CTSB (cathepsin B), which is frequently used as a marker of functional lysosomes.^29^ Gefitinib and AZD9291 effectively reduced the processing of pro-CTSB to mature CTSB (Fig. 7a–c; Supplementary Fig. 5A–C), indicating a defect in lysosomal function after gefitinib and AZD9291 treatment. Next, we used the LysoTracker probe to trace acidic lysosomes.^30^ Interestingly, gefitinib and AZD9291 evidently decreased LysoTracker Blue staining similar to CQ,^31^ indicating a reduction in the acidity of the lysosomal lumen (Fig. 7d; Supplementary Fig. 5D). To further confirm this finding, we used another dye, AO (acridine orange), that emits green fluorescence in the cytosol in the monomeric form but aggregates into the protonated form to generate bright red fluorescence when trapped in acidic lysosomes.^32^ Similar to CQ and BafA1, gefitinib and AZD9291 significantly decreased the number of cytoplasmic AO (red) dots (Fig. 7e; Supplementary Fig. 5E). Since gefitinib and AZD9291 are selective inhibitors of EGFR and function by binding to the tyrosine kinase domain of EGFR, we next explored how gefitinib and AZD9291 attenuate lysosome acidification. Interestingly, we found that EGFR knockdown markedly decreased SQSTM1 expression (Supplementary Fig. 5F), suggesting that the attenuation of autophagic degradation by gefitinib and AZD9291 might be independent of their ability to inhibit EGFR signaling. A previous high content screening assay identified gefitinib as a lysosomotropic agent for its alkalinity.^31^ The basic pKa values of gefitinib and AZD9291 calculated with ChemAxon are 6.85 and 8.87, respectively. Thus, we investigated whether gefitinib and AZD9291 can accumulate in lysosomes similar to CQ, which inhibits lysosome acidification due to its intrinsic alkalinity. To ascertain whether gefitinib and AZD9291 can translocate to lysosomes, we constructed fluorescein-labeled gefitinib with rhodamine B and named it Gefi-RB (Supplementary Fig. 6A, B). Notably, most Gefi-RB colocalized with lysosomes labeled with LysoTracker Blue, suggesting that gefitinib probably disrupts lysosomal function by accumulating in the lysosome lumen (Fig. 7f). Furthermore, genetic knockdown of EGFR expression had little effect on the lysosomal translocation of Gefi-RB (Fig. 7g). Consistently, knockdown of EGFR expression played a limited role in the repression of lysosomal acidification by gefitinib and AZD9291 (Supplementary Fig. 5G). Altogether, these results indicate that gefitinib and AZD9291 repress lysosomal function by disturbing lysosomal lumen acidification through their intrinsic alkalinity, independent of EGFR inhibition.

### Alkaline-neutralizing modification of gefitinib increases the sensitivity of NSCLC cells

**Fig. 8:**
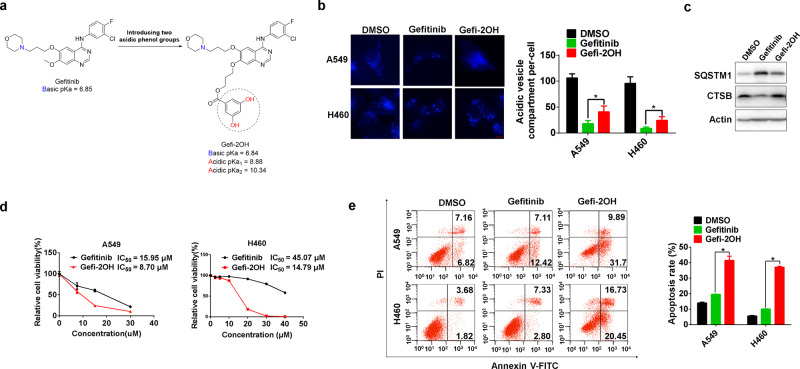
Acidic transformation of gefitinib increases the sensitivity of NSCLC cells. **a** Design strategy for the alkaline-neutralizing gefitinib analog (Gefi-2OH). The pKa values of gefitinib and Gefi-2OH were estimated by a freely available package, Marvin by ChemAxon, based on the Hammett-Taft approach. **b** A549 and H460 cells were treated with DMSO, gefitinib, or Gefi-2OH for 12 h and then incubated with LysoTracker Blue for 2 h. Fluorescence microscopy was used to detect the number of acidic vesicles. Scale bar: 10 μm. The quantification of acidic vesicle compartments per cell is expressed as the mean ± SD (*n* = 10 cells, three independent experiments). Asterisks indicate significant differences (Student’s *t* test, *p* < 0.05). ND, not detected. **c** H460 cells were treated with gefitinib or Gefi-2OH for 24 h, and the expression of SQSTM1, CTSB, and LC3 was measured by western blotting. **d** A549 and H460 cells were treated with different concentrations of gefitinib or Gefi-2OH for 48 h, and then, the cell viability was measured by MTS assay. **e** A549 and H460 cells were treated with gefitinib or Gefi-2OH for 24 h. The annexin V-PI staining assay was performed after the indicated treatment, and the relative percentage of apoptotic cells is summarized

To further confirm our findings, we synthesized a novel alkaline-neutralizing gefitinib analog. Based on the previously reported crystal structure of EGFR in complex with gefitinib (PDB ID: 2ITY),^33^ the methoxy group in the 7-position of the quinazoline oriented toward the solvent area was chosen for modification to minimize the influence on EGFR inhibition. In brief, we introduced two phenolic hydroxyl groups into the structure of gefitinib to make it less basic and named this derivate Gefi-2OH (Fig. 8a; Supplementary Fig. 7A, B). As expected, Gefi-2OH and gefitinib had a similar inhibitory effect on EGFR signaling (Supplementary Fig. 7C). Moreover, in contrast to gefitinib, Gefi-2OH lost the ability to alkalize lysosomes and thus was incapable of inhibiting mature CTSB expression and SQSTM1 degradation (Fig. 8b, c). Interestingly, the inhibition of cell viability and the activation of apoptosis by Gefi-2OH were significantly potentiated compared with parental gefitinib (Fig. 8d, e). In summary, these data indicate that lysosomal repression by gefitinib is attributed to its physicochemical properties and that modification to alleviate alkalinity enhances the antitumor effect of gefitinib.

## Discussion

The development of EGFR TKIs has reformed the clinical management of lung cancer. However, the war on lung cancer is still far from over. Lung cancer remains the most frequent cause of cancer-associated death worldwide. Resistance to EGFR TKIs is currently the biggest challenge. While new generations of EGFR TKIs have been developed, acquired resistance continues to develop, indicating the urgent need for alternative strategies, such as targeting the intrinsic protective responses to EGFR TKIs. In this study, we report that targeting autophagy-related molecules might be valuable for overcoming EGFR TKI resistance.

As a stress response, autophagy has been reported to play a complicated role in the initiation and progression of human cancers, including roles in the resistance to chemotherapy and targeted therapeutics.^34,35^ While autophagy deficiency resulting from the genetic knockout of genes essential for autophagy eventually promotes tumorigenesis in mice,^36^ autophagy can be activated in response to various stresses to protect cells from stress-induced cell death, thus promoting tumorigenesis and conferring drug resistance. By providing nutrients and building blocks essential for tumor growth, enhanced autophagy can benefit tumor cells in the competitive landscape of the hostile microenvironment.^37^ Therefore, the anti-malaria drug CQ and its derivatives, such as HCQ, have been proposed to be valuable therapeutics for human cancers. However, CQ represses the proliferation of ATG7-deficient cancer cells,^38^ indicating that CQ might exert its tumor inhibitory effect independent of autophagy inactivation. Moreover, clinical trials of CQ alone or in combination reported limited antitumor effects, indicating a complicated function of CQ. For example, it would be interesting to ascertain whether the accumulation of SQSTM1 resulting from the CQ-mediated inhibition of autolysosome function contributes to the limited clinical efficacy of CQ. SQSTM1 has tumor-promoting capacity in addition to its well-known pro-autophagy function by activating Nrf2, mTORC1, and NFκB signaling pathways.^39,40^ In line with this, we confirmed that knockdown of SQSTM1 expression further enhanced the inhibition of cell proliferation by CQ (data not shown).

Herein, we found that SQSTM1 accumulation conferred resistance to gefitinib and AZD9291. Depleting SQSTM1 sensitized NSCLC cells to gefitinib and AZD9291 by inducing apoptosis both in vitro and in vivo. In addition, longer durations of gefitinib and AZD9291 treatment caused a slight increase in SQSTM1 mRNA, although the mechanism was not identified. There is evidence that EGFR TKIs can increase reactive oxygen species (ROS), which would activate Nrf2, a transcription factor for various reductases and SQSTM1.^23,41,42^ Consequently, oxidative stress might be involved in the slight upregulation of SQSTM1 mRNA triggered by EGFR TKIs. Therefore, targeting SQSTM1 might be a potential therapeutic strategy to improve the efficacy of gefitinib and AZD9291 in patients with advanced NSCLC. It has been reported that SQSTM1 is able to regulate apoptosis-related signaling. For example, SQSTM1 can compete with Nrf2 for binding to KEAP1, thus protecting Nrf2 from ubiquitination-dependent proteasomal degradation.^19,43^ In addition, SQSTM1 can interact with TRAF-6 (TNF receptor-associated factor 6), resulting in activation of the NFκB signaling pathway.^20^ SQSTM1 also participates in DDR (DNA damage repair) by regulating DDR-relevant factors.^44^ In line with these findings, SQSTM1 contributes to resistance to several antitumor drugs, including cisplatin,^45^ proteasome inhibitors,^46^ and methotrexate.^47^ Taken together, these data indicate that targeting SQSTM1 accumulation may represent a new strategy to overcome drug resistance. Meanwhile, SQSTM1 is prone to form aggregates or sequestosomes.^48^ SQSTM1 is a common constituent of cytoplasmic aggregates in various diseases such as Alzheimer’s disease and hepatocellular carcinoma.^49,50^ Nevertheless, clinical trials are warranted to confirm the value of targeting SQSTM1 accumulation in these disorders.

Much to our surprise, gefitinib and AZD9291 blocked rather than activated autophagy flux, although they effectively repressed PI3K/mTOR signaling pathway. Gefitinib and AZD9291 actually impaired lysosomal function and prevented the autolysosomal degradation of SQSTM1, similar to CQ and BafA1, which impair the acidification of lysosomes. Interestingly, gefitinib and AZD9291 attenuated lysosome acidification due to their intrinsic alkalinity. Both gefitinib (pKa = 6.85) and AZD9291 (pKa = 8.87) have a ClogP > 2 and a basic pKa between 6.5 and 11, which indicates that they are lysosomotropic. Chemical modification of gefitinib to alleviate alkalinity evidently potentiated its antitumor effect. Theoretically, acidic modification of AZD9291, a more alkaline EGFR TKI than gefitinib, will also lead to improved therapeutic efficacy. Hence, this new finding could inspire new strategies for improving the clinical efficacy of gefitinib and AZD9291 by modifying their physicochemical properties. Future works evaluating the effects of chemically modified other basic TKIs on NSCLC and other cancer cells would further promote the optimization of current TKIs and improve the outcomes of various cancer patients undergoing TKI treatment.

In summary, gefitinib and AZD9291 impaired lysosomal function owing to their intrinsic alkalinity, thereby blocking autophagic degradation and resulting in the accumulation of tumor-promoting SQSTM1. Knockdown of SQSTM1 expression significantly sensitized NSCLC cells to gefitinib and AZD9291 both in vitro and in vivo. Chemically modified gefitinib lacking alkalinity displayed enhanced inhibitory effects on NSCLC. Therefore, targeting SQSTM1 accumulation or chemically modifying gefitinib and AZD9291 could be new strategies to overcome or delay gefitinib and AZD9291 resistance.

## Supplementary information

Supplementary Information
