# Supplementary material for: EGFR TKIs impair lysosome-dependent degradation of SQSTM1 to compromise the effectiveness in lung cancer
Source: Signal Transduct Target Ther. 2019 Jul 12;4:25. doi: 10.1038/s41392-019-0059-4 (PMC6799834; doi:10.1038/s41392-019-0059-4)
Supplement: Supplementary file 1 — Supplementary Information [file 41392_2019_59_MOESM1_ESM.docx]

**Supplementary Information**

**EGFR TKIs impair lysosome-dependent degradation of SQSTM1 to compromise the effectiveness in lung cancer**

Lixian Yang^1,#^, Shilong Ying^1,#^, Shiman Hu^1^, Xiangtong Zhao^1^, Muchun Li^1^, Miaoqin Chen^1^, Yiran Zhu^1^, Ping Song^2^, Liyuan Zhu^1^, Tingting Jiang^1^, Huimin An^3^, Neelum Aziz Yousafzai^2^, Wenxia Xu^1^, Zhiguo Zhang^4^, Xian Wang^2^, Lifeng Feng^1,^*, Hongchuan Jin^1,^*

^1^Laboratory of Cancer Biology, Key Lab of Biotherapy in Zhejiang, Sir Run Run Shaw Hospital, Medical School of Zhejiang University, Hangzhou, China; ^2^Department of Medical Oncology, Key Lab of Biotherapy in Zhejiang, Sir Run Run Shaw Hospital, Medical School of Zhejiang University, Hangzhou, China; ^3^Department of Pathology, Sir Run Run Shaw Hospital, Medical School of Zhejiang University, Hangzhou, China; ^4^Key Laboratory of Biomass Chemical Engineering of Ministry of Education, College of Chemical and Biological Engineering, Zhejiang University, Hangzhou, China.

**Running title**: Stabilization of SQSTM1 by EGFR TKIs

#: These two authors contributed to this work equally.

***Correspondence to**: Dr. Lifeng Feng, [lffeng@zju.edu.cn;](mailto:lffeng@zju.edu.cn;) Dr. Hongchuan Jin, [jinhc@zju.edu.cn](mailto:jinhc@zju.edu.cn)

This file contains Supplemental Materials and Methods and Figure S1-S7

**Supplemental Materials and Methods**

**Materials**

All reagents were purchased from commercial sources and were used without further purification. Reactions were monitored by thin-layer chromatography (TLC) on 0.25 mm silica gel plates with fluorescent indicator (GF254) and visualized under UV light. ^1^H-NMR were recorded on Bruker Avance 400 spectrometer (400 MHz, Bruker, German), Bruker DMX-500 (500 MHz, Bruker, German), or Agilent DirectDrive2 (600 MHz, Agilent, California, USA). Electrospray ionization mass spectra were obtained on Agilent-1100 series mass spectrometer (Agilent, California, USA). MALDI-TOF mass spectra was recorded on a GCT Premier (WATERS, Massachusetts, USA).

**Chemical synthesis of fluorescein-labelled Gefitinib (Gefi-RB)**

**Synthesis of demethylated Gefitinib (1):** Demethylated Gefitinib was synthesized according to the methods reported previously with modifications [1]. Briefly, to a stirred solution of Gefitinib (90 mg, 0.2 mmol) in methanesulfonic acid (5 mL) was added L-methionine (49.5 mg, 0.33 mmol). The reaction mixture was heated to 165 ^o^C and stirred for about 25–30 hrs. The completion of the reaction was monitored by TLC. After the completion of the reaction, cooled to RT, quenched in ice-water and pH was adjusted to 7 with 40% NaOH aqueous solution. The mixture was extracted with EtOAc (3×20 mL), washed with water (10 mL) and evaporated to remove the solvents under vacuum. Purification using column chromatography (DCM/MeOH = 20/1 to 4/1) gave compound **1** (52% yield) as a yellowish powder. ^1^H-NMR (CD_3_OD, 400MHz) δ: 8.367 (s, 1H, H-2), 7.964 (dd, *J_1_* = 2.8 Hz, *J_2_* = 6.8 Hz, 1H, H-8), 7.762 (s, 1H, H-5), 7.616-7.656 (m, 1H, H-2’), 7.230 (t, *J* = 8.8 Hz, 1H, H-6’), 7.033 (s, 1H, H-5’), 4.267 (t, *J* = 6.0 Hz, 2H, -NCH_2_CH_2_CH_2_O-), 3.783 (t, *J* = 4.8 Hz, 4H, -OCH_2_×2), 2.871 (t, *J* = 7.2 Hz, 2H, -NCH_2_CH_2_CH_2_O-), 2.784 (s, 4H, -NCH_2_×2), 2.145-2.208 (m, 2H, -NCH_2_CH_2_CH_2_O-). MS (ESI) m/z: 432.97 (M+1)^+^, calculated for C_21_H_22_ClFN_4_O_3_: 432.14.

**Synthesis of the intermediate 3**: The title compound was prepared according to the reference [2]. To a solution of rhodamine B (960 mg, 2 mmol) in 1,2-dichloroethane (DCE, 10 mL) was added phosphorus oxychloride (0.56 mL, 6 mmol) dropwise. The solution was refluxed for 4 hrs. The reaction mixture was cooled and concentrated under reduced pressure to give the rhodamine B acid chloride **2**, which was used in the next step without further purification. The crude acid chloride was dissolved in DCM (20 mL) and the solution was slowly added 3-bromopropylamine hydrobromide (219 mg, 1 mmol) and trimethylamine (0.556 mL, 4 mmol) in DCM (2 mL) dropwise. This solution was allowed to stir for 24 hrs at ambient temperature, which was then transferred to a separatory funnel with an additional 20 mL of DCM. The organic layer was washed with water (2 x 20 mL), and dried over MgSO_4_. The solvent was concentrated in vacuum. Purification using column chromatography (DCM/MeOH = 30/1 to 20/1) gave intermediate **3** (55% yield) as a pale red powder. ^1^H-NMR (CDCl_3_, 400MHz) δ: 7.844 (m, 1H, Ar-6), 7.425-7.468 (m, 2H, Ar-4, Ar-5), 7.082-7.103 (m, 1H, Ar-3), 6.421 (d, *J* = 8.8 Hz, 4H, Ar’-2×2, Ar’-3×2), 6.303 (d, *J* = 7.6 Hz, 4H, Ar’-5×2), 3.344 (q, *J* = 7.2 Hz, 8H, Ar’-NCH_2_×4), 3.222 (t, *J* = 7.2 Hz, 2H, -NCH_2_CH_2_CH_2_Br), 3.148 (t, *J* = 7.2 Hz, 2H, -NCH_2_CH_2_CH_2_Br), 1.666-1.736 (m, 2H, -NCH_2_CH_2_CH_2_Br), 1.171 (t, *J* = 7.2 Hz, 12H, Ar’-NCH_2_CH_2_×4). MS (ESI) m/z: 562.27 (M+1)^+^, 564.27 (M+3)^+^, calculated for C_31_H_36_BrN_3_O_2_: 561.20.

**Synthesis of targeted fluorescein-labelled Gefitinib (Gefi-RB):** To a solution of compound **1** (43.2 mg, 0.1 mmol) in DMF (5 mL) was added K_2_CO_3_ (55.2 mg, 0.4 mmol) and the obtained mixture was stirred at room temperature for 0.5 hr. Then Intermediate **3** (84.3 mg, 0.15 mmol) was added. The reaction mass was heated to 80 ^o^C and stirred overnight. The resultant mixture was extracted with DCM and the obtained organic phase was washed with H_2_O and brine, dried over MgSO_4_, and concentrated in vacuum. Purification using column chromatography (DCM/MeOH = 20/1) gave compound **Gefi-RB** (46% yield) as a white powder. ^1^H-NMR (CDCl_3_, 400MHz) δ: 8.553 (s, 1H, H-2), 8.011 (dd, *J_1_* = 2.4 Hz, *J_2_* = 6.8 Hz, 1H, H-8), 7.844 (dd, *J_1_* = 2.8 Hz, *J_2_* = 5.6 Hz, 1H, Ar-6), 7.720-7.759 (m, 1H, Ar-4), 7.481 (s, 1H, H-5), 7.345 (t, *J* = 5.6 Hz, 1H, H-2’), 7.345 (d, *J* = 6.8 Hz, 1H, H-6’), 7.124 (t, *J* = 8.8 Hz, 1H, H-5’), 7.088 (dd, *J_1_* = 2.4 Hz, *J_2_* = 6.0 Hz, 1H, Ar-5), 6.996 (s, 1H, Ar-3), 6.420 (s, 1H, Ar’-2), 6.398 (s, 1H, Ar’’-2), 6.359 (d, *J* = 2.4 Hz, 2H, Ar’-3, Ar’’-3), 6.254 (dd, *J_1_* = 2.4 Hz, *J_2_* = 8.8 Hz, 2H, Ar’-5, Ar’’-5), 4.090 (t, *J* = 6.0 Hz, 2H, -OCH_2_CH_2_CH_2_NC=O), 3.852 (d, *J* = 0.8 Hz, 6H, 7-OCH_2_, morpholinyl-O-CH_2_×2), 3.379 (t, *J* = 6.8 Hz, 2H, -OCH_2_CH_2_CH_2_NC=O), 3.305 (q, *J* = 7.2 Hz, 8H, Ar’-NCH_2_×2, Ar’’-NCH_2_×2), 2.860 (s, 2H, 6-OCH_2_CH_2_CH_2_), 2.818 (s, 4H, morpholinyl-N-CH_2_×2), 2.107-2.170 (m, 2H, -OCH_2_CH_2_CH_2_NC=O), 1.689-1.743 (m, 2H, 6-OCH_2_CH_2_CH_2_), 1.1463 (t, *J* = 7.2 Hz, 12H, Ar’-NCH_2_CH_2_×2, Ar’’-NCH_2_CH_2_×2). MS (ESI) m/z: 914.65 (M+1)^+^, 912.49 (M-1)^-^, calculated for C_52_H_57_ClFN_7_O_5_: 913.41.

**Chemical synthesis of compound Gefitinib-2OH (Gefi-2OH)**

**Synthesis of intermediate 4:** To a solution of 3, 5-dimethoxybenzoyl chloride (2 g, 10 mmol) in 20 mL of DCM was added of triethylamine (1.5 mL, 15 mmol) and 3-bromo-1-propanol (1.07 mL, 5 mmol) and the mixture was stirred for 5 hrs at ambient temperature. The reaction is rinsed with water, dried (MgSO_4_), filtered and purified by flash column chromatography with 25% EtOAc in hexane to obtain intermediate **4** (80% yield) as a colorless oil.

**Synthesis of intermediate 5:** Intermediate **4** (604 mg, 2 mmol) was dissolved in DCM (10 mL). Aluminum trichloride (1333 mg, 10 mmol) was added portion-wise firstly, then sodium iodide (150 mg, 1 mmol) was added into subsequently. The reaction mixture was stirred for 6-12 hrs at room temperature. At the end, the reaction was quenched with saturated aqueous NH_4_Cl solution (1 mL): the aqueous layer was extracted twice with DCM (10 mL) and the organic layers were collected, washed with saturated aqueous NaCl solution (10 mL) and dried over MgSO_4_. The solution was filtrated and the solvent was evaporated under vacuum. The crude was subjected to flash chromatography (DCM/MeOH = 100/1 to 20/1) to afford the demethylated product **5** (30% yield) as a yellowish oil. ^1^H-NMR (CDCl_3_, 600MHz) δ: 7.087 (d, *J* = 2.4 Hz, 2H, H-2, H-6), 6.572 (t, *J* = 2.4 Hz, 1H, H-4), 5.486 (s, 2H, -OH×2), 4.449 (t, *J* = 6.0 Hz, 2H, -OCH_2_CH_2_CH_2_Br), 3.676 (t, *J* = 6.6 Hz, 2H, -OCH_2_CH_2_CH_2_Br), 2.212 (t, *J* = 6.0 Hz, 2H, -OCH_2_CH_2_CH_2_Br).

**Synthesis of intermediate 6:** The diphenol intermediate **5** (274 mg, 1 mmol) and pyridinium p-toluenesulfonate (50.2 mg, 0.2 mmol) were stirred at room temperature for 0.5 hr in DCM (10 mL), after which 3,4-dihydro-α-pyrane (0.91 mL, 10 mmol), dissolved in 5 mL of DCM, was added dropwise at the same temperature. The reaction mixture was stirred at room temperature for 4-6 hrs and then was washed twice with water, dried, and evaporated in vacuo. The obtained crude product was purified by column chromatography (DCM/MeOH = 100/3 to 100/5) to give intermediate **6** (63% yield) as a colorless oil. ^1^H-NMR (CDCl_3_, 500MHz) δ: 7.348 (t, *J* = 2.5 Hz, 2H, H-2, H-6), 6.984 (q, *J* = 2.5 Hz, 1H, H-4), 5.435-5.463 (m, 2H, -O-pyranyl-H-2×2), 4.447 (t, *J* = 6.0 Hz, 2H, -OCH_2_CH_2_CH_2_Br), 3.886 (td, *J_1_* = 2.5 Hz, *J_2_* = 10.5 Hz, 2H, -O-pyranyl-H-6×2), 3.688 (t, *J* = 6.5 Hz, 2H, -OCH_2_CH_2_CH_2_Br), 3.596-3.632 (m, 2H, -O-pyranyl-H-6×2), 2.196-2.246 (m, 2H, -OCH_2_CH_2_CH_2_Br), 1.958-2.016 (m, 2H, -O-pyranyl-H-3×2), 1.844-1.894 (m, 4H, -O-pyranyl-H-3×2, -O-pyranyl-H-4×2), 1.644-1.712 (m, 4H, -O-pyranyl-H-4×2, -O-pyranyl-H-5×2), 1.584-1.613 (m, 2H, -O-pyranyl-H-5×2).

**Synthesis of alkaline-neutralizing Gefitinib analogue (Gefi-2OH):** To a solution of compound **1** (43.2 mg, 0.1 mmol) in DMF (5 mL) was added K_2_CO_3_ (55.2 mg, 0.4 mmol) and the obtained mixture was stirred at room temperature for 0.5 hr. Then Intermediate **6** (88.4 mg, 0.2 mmol) and NaI (15 mg, 0.1 mmol) was added. The reaction mass was heated to 80 ^o^C and stirred overnight. The reaction mixture was carefully poured into water (5 mL), and extracted with EtOAc (3x20 mL). The organic layer was then dried over MgSO_4_ and evaporated to remove the solvents. The obtained crude product was purified by column chromatography (DCM/MeOH = 100/3 to 100/5) to produce the THP-protected intermediate (41% yield) as a white powder. This intermediate was re-dissolved in 5 mL EtOH, and 10% HCl aqueous solution (1 mL) was added to deprotect the THP group. The solution was stirred for 1 hr. Then it was neutralized with saturated sodium carbonate aqueous and extracted with EtOAc (4x10 mL). The organic solution was dried over MgSO_4_ and evaporated. The residue was purified by column chromatography (DCM/MeOH = 30/1 to 20/1) to yield the target compound Gefi-2OH (30% yield) as a white powder. ^1^H-NMR (DMSO-d_6_, 600MHz) δ: 8.471 (s, 1H, H-2), 8.116 (d, *J* = 4.8 Hz, 1H, H-8), 7.849 (s, 1H, H-5), 7.785 (s, 1H, H-2’), 7.410 (t, *J* = 9.0 Hz, 1H, H-6’), 7.222 (s, 1H, H-5’), 6.803 (d, *J* = 2.4 Hz, 2H, H-2’’, H-6’’), 6.410 (t, *J* = 2.4 Hz, 1H, H-4’’), 4.400 (t, *J* = 6.0 Hz, 2H, O=COCH_2_), 4.280 (t, *J* = 6.0 Hz, 2H, O=COCH_2_CH_2_CH_2_), 4.177 (s, 2H, -NCH_2_CH_2_CH_2_O-), 3.556 (s, 4H, -OCH_2_×2), 2.475 (t, *J* = 1.8 Hz, 2H, -NCH_2_CH_2_CH_2_O-), 2.359 (s, 4H, -NCH_2_×2), 2.218 (m, *J* = 6.0 Hz, 2H, O=COCH_2_CH_2_CH_2_), 1.983 (m, *J* = 7.8 Hz, 2H, -NCH_2_CH_2_CH_2_O-). MALDI-TOF MS m/z: 627.475 (M+1)^+^, calculated for C_31_H_32_ClFN_4_O_7_: 626.19.

**References:**

[1]. Chandregowda V, Kush AK, Chandrasekara Reddy G. Synthesis and in vitro antitumor activities of novel 4-anilinoquinazoline derivatives. Eur J Med Chem. 2009 Jul;44(7):3046-55. doi: 10.1016/j.ejmech.2008.07.023. PubMed PMID: 18771819.

[2]. Best QA, Liu C, van Hoveln PD, et al. Anilinomethylrhodamines: pH sensitive probes with tunable photophysical properties by substituent effect. J Org Chem. 2013 Oct 18;78(20):10134-43. doi: 10.1021/jo401323g. PubMed PMID: 24050117; PubMed Central PMCID: PMCPMC3992268.

**Supplemental Figure and Figure legends**

**
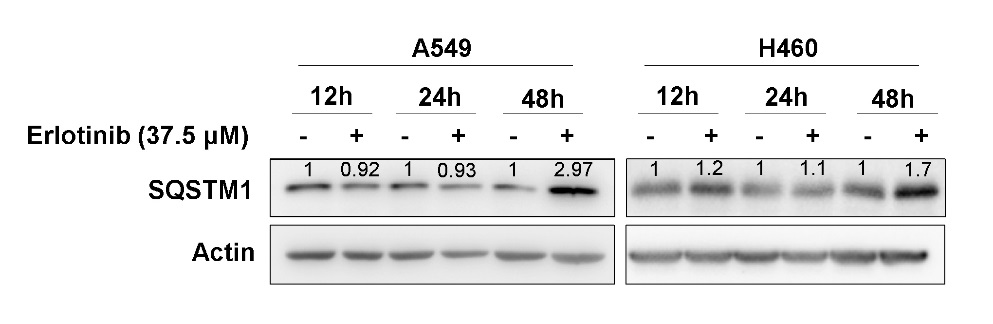
**

**Supplemental Figure 1 Erlotinib upregulates SQSTM1 in NSCLC.** A549 and H460 were treated with Erlotinib for indicated time, and the expression of SQSTM1 was measured by Western blotting. Relative SQSTM1 expression (SQSTM1/Actin) was quantified and normalized to the relative SQSTM1 expression in the control.

**
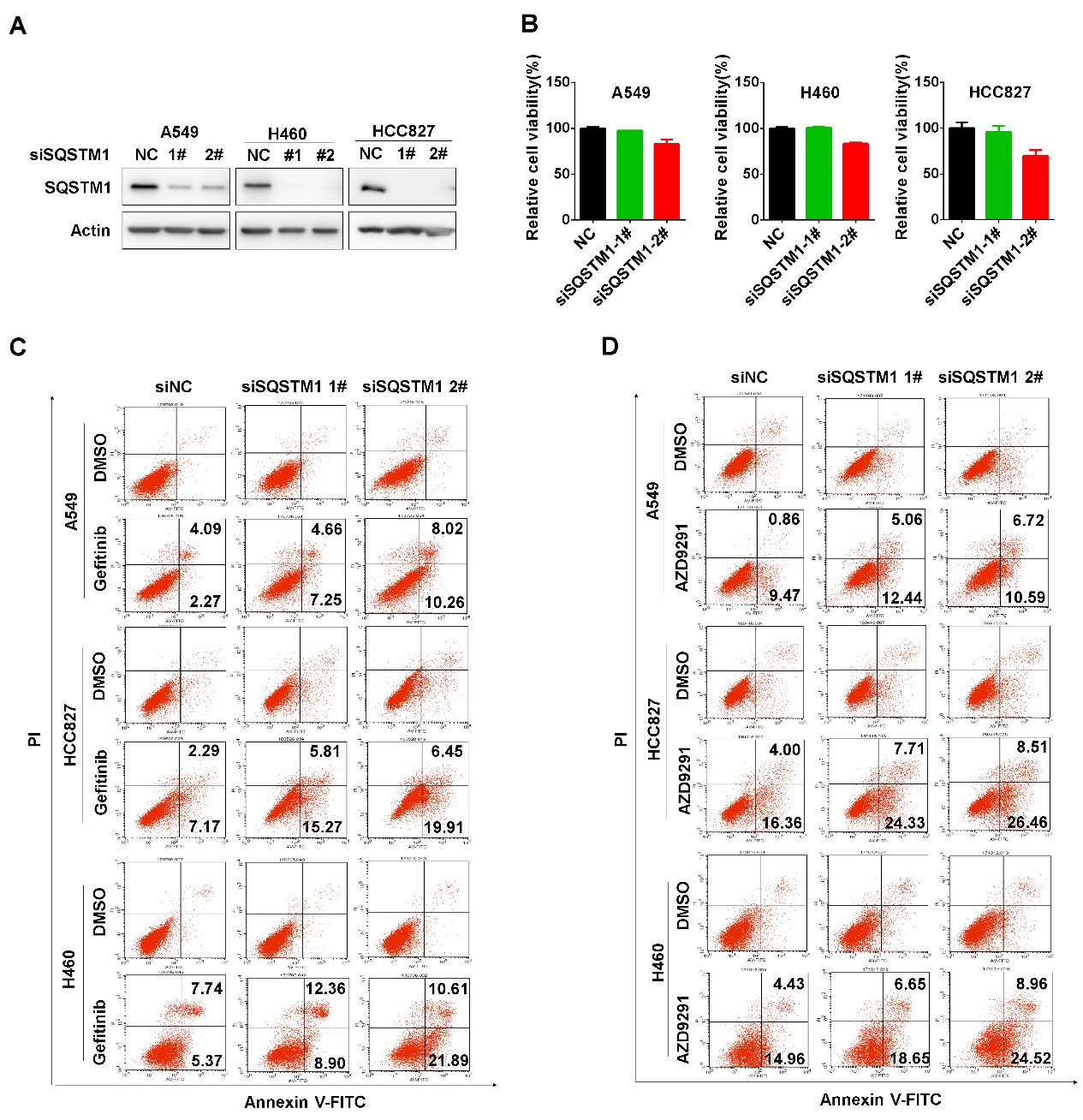
**

**Supplemental figure 2 SQSTM1 knock-down sensitized NSCLC to Gefitinib and AZD9291.** (**A**) A549, HCC827 and H460 cells were transfected with siNC or siSQSTM1 for 48 hrs and then SQSTM1 knockdown was confirmed by Western blotting. (**B**) A549, HCC827 and H460 cells were transfected with siNC or siSQSTM1 for 48 hrs before viability analysis by MTS assay. (**C-D**) The annexinV-PI staining assay was performed after indicated treatments and the represented data was shown.


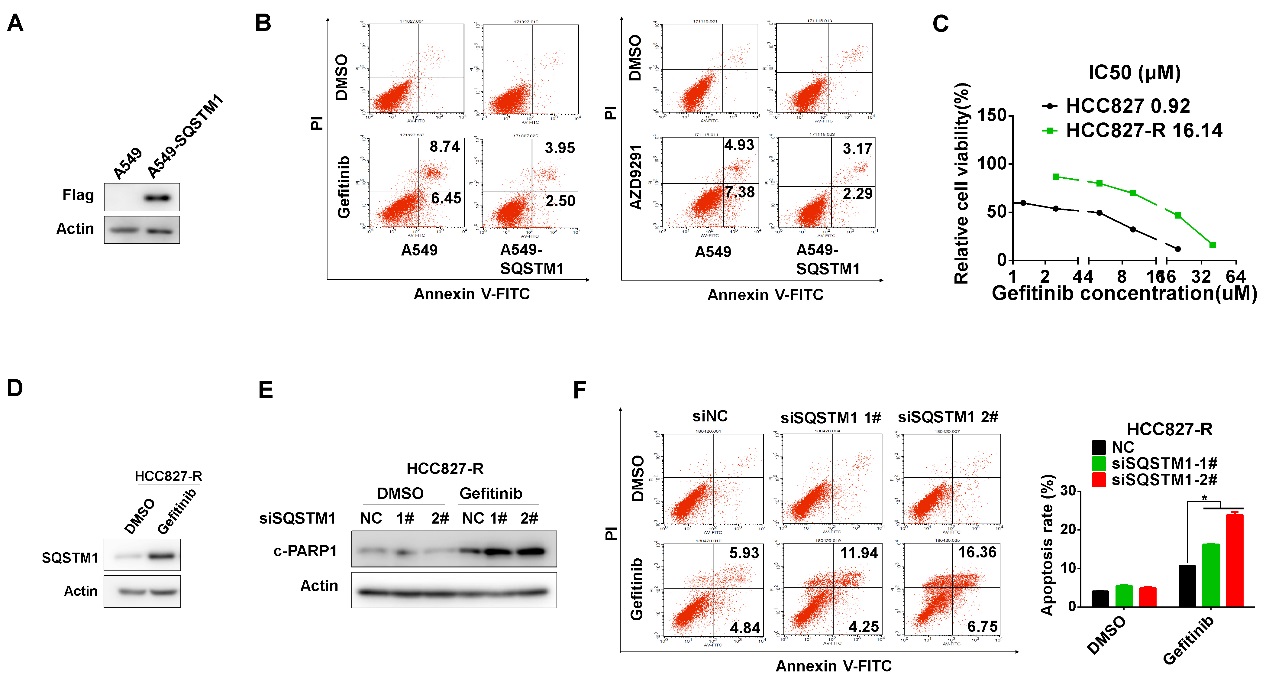


**Supplemental figure 3 SQSTM1 overexpression confer Gefitinib and AZD9291 resistance *in vitro* and *vivo*.** (**A**) Western blotting was performed to measure the expression of Flag-SQSTM1 in A549 and A549-SQSTM1 cells. (**B**) A549 and A549-SQSTM1 cells were treated with Gefitinib or AZD9291 for 48 hrs and annexinV-PI staining assay was performed after indicated treatments. The represented data was shown. (**C**) HCC827 and HCC827-R cells were treated with Gefitinib for 48 hrs. Cell viability were detected by MTS assay and IC50 (half-maximal inhibitory concentration) was shown. (**D**) HCC827-R cell line was treated with Gefitinib for 24 hrs and SQSTM1 expression was measured by Western blotting. (**E**) HCC827-R cell was treated with Gefitinib after siSQSTM1 transfection for 48 hrs. Western blotting was applied to measure c-PARP1 expression. (**F**) HCC827-R cell was treated with Gefitinib after SQSTM1 knocking down and annexinV-PI staining assay was performed 72 hrs later. The relative percentage of apoptotic cells was summarized (right panel). Student’s *t* test was used for the statistical analysis. Asterisks indicate statistical significance (*p*<0.05).

**
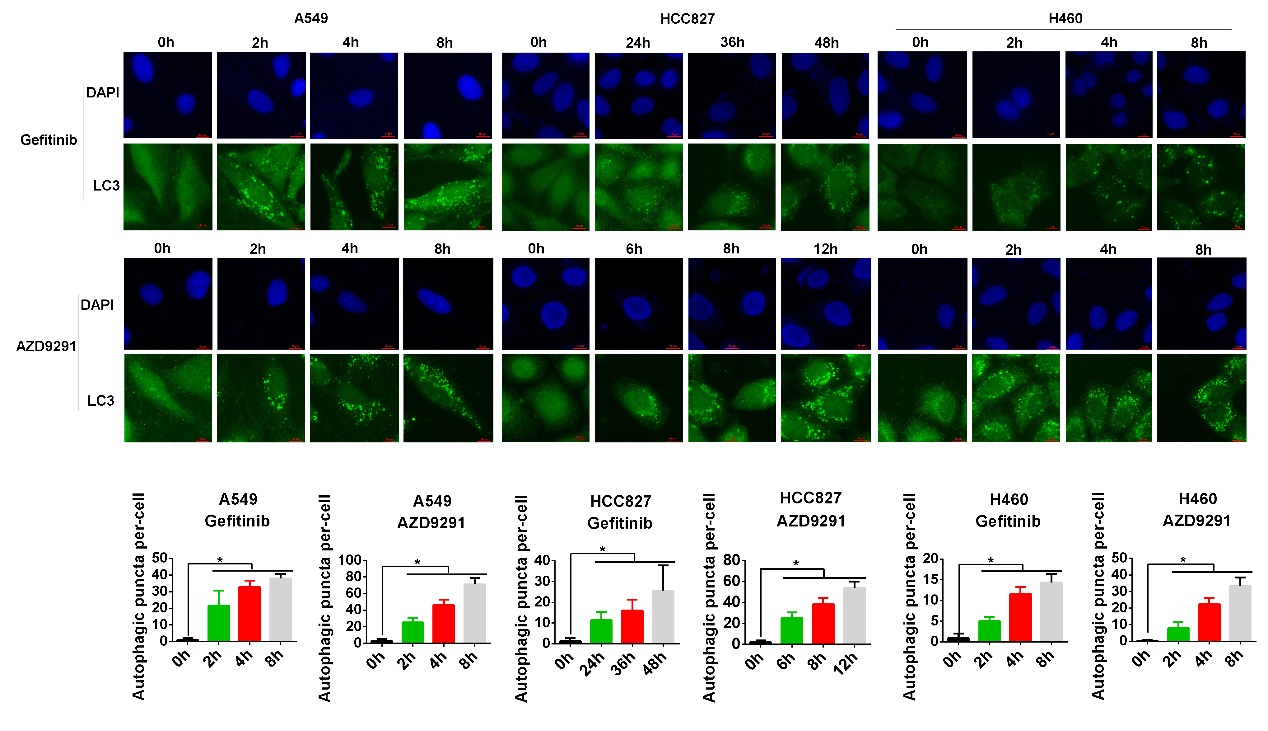
**

**Supplemental figure 4 Gefitinib and AZD9291 increased autophagsome formation in NSCLC cells.** A549, HCC827 and H460 cells were treated with Gefitinib or AZD9291 for indicated time and immunofluorescence microscopy was performed to detect the puncta of LC3 as the representation for autophagic puncta. Scale bar: 10μM. Quantification of autophagic puncta is expressed as Mean±SD, n=10 cells of 3 independent experiments. Asterisks indicates statistical difference (Student’s *t* test, *p*<0.05).

**
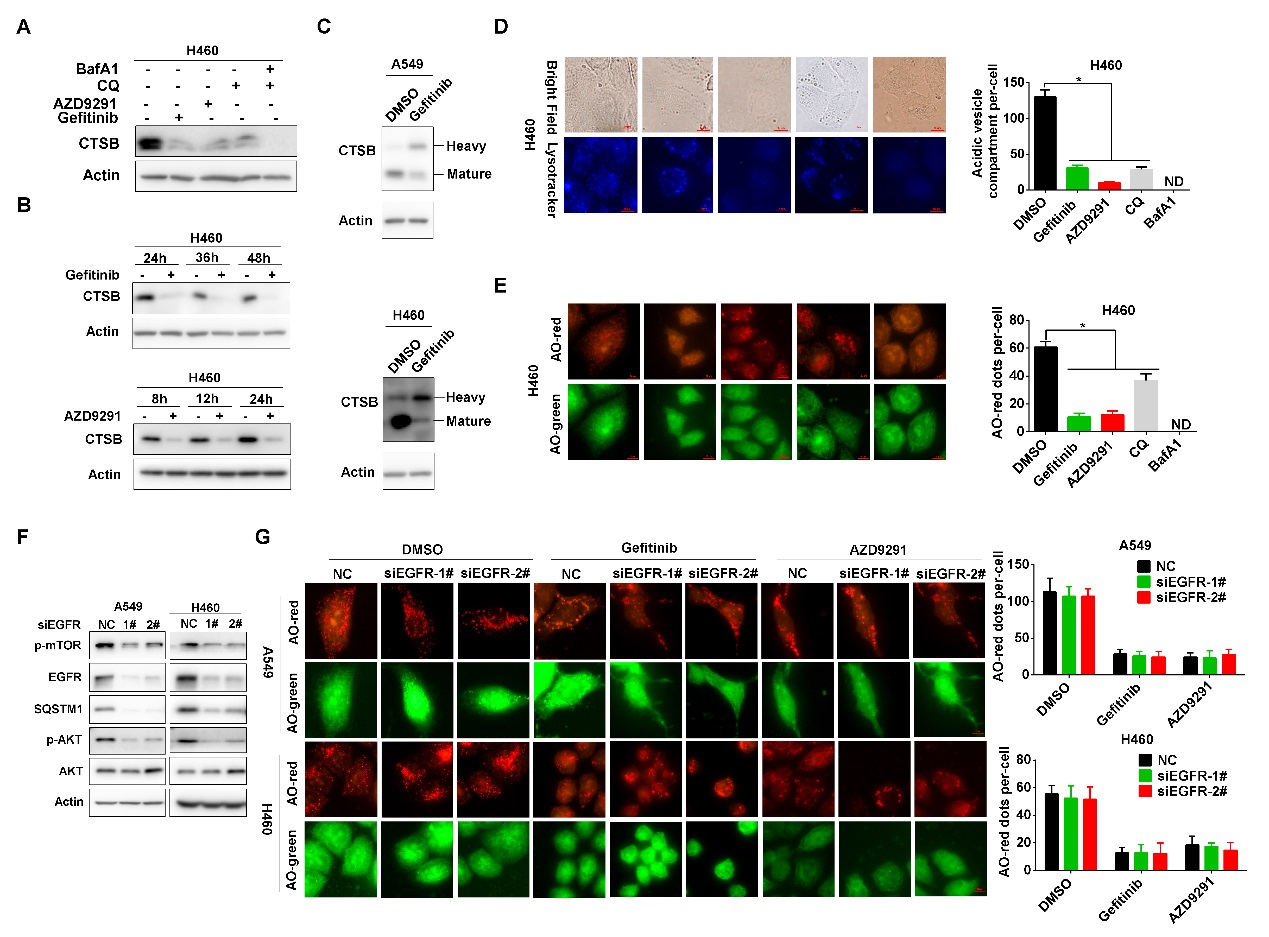
**

**Supplemental figure 5** (A) H460 cell was treated with Gefitinib, AZD9291, CQ or BafA1 for 24 hrs and the expression of mature CTSB was assessed by Western blotting. (B) H460 cell was treated with Gefitinib or AZD9291 for indicated time and the expression of mature CTSB was assessed by Western blotting. (C) A549 and H460 were treated with Gefitinib for 24 hrs and the expression of proform and mature CTSB was assessed by Western blotting. (D) H460 cell was treated with DMSO, Gefitinib, AZD9291, CQ or BafA1 for 12 hrs and then incubated with LysoTracker Blue for 2 hrs. Fluorescent microscopy was used to detect the number of acidic vesicles. Scale bar: 10μM. Quantification of acidic vesicle compartment per-cell is expressed as Mean±SD, n=10 cells of 3 independent experiments. Asterisks indicates statistical difference (Student’s t test, p<0.05) and ND represents not detected. (E) H460 cell was treated with DMSO, Gefitinib, AZD9291, CQ or BafA1 for 12 hrs and then incubated with AO for 15 min. Fluorescent microscopy was used to detect the number of AO-red dots. Scale bar: 10μM. Quantification of AO-red dots per-cell is expressed as Mean±SD, n=10 cells of 3 independent experiments. Asterisks indicates statistical difference (Student’s t test, p<0.05) and ND represents not detected. (F) A549 and H460 have been knocked down EGFR for 36hrs and the expression of p-mTOR, EGFR, SQSTM1, p-AKT and AKT were detected by Western blotting. (G) A549 and H460 cells were treated with Gefitinib or AZD9291 for 12hrs after knocking down EGFR for 24hrs, and then incubated with AO for 15 min. Fluorescent microscopy was used to detect the number of AO-red dots. Scale bar: 10μM. Quantification of AO-red dots per-cell is expressed as Mean±SD, n=10 cells of 3 independent experiments. Asterisks indicates statistical difference (Student’s t test, p<0.05).

**
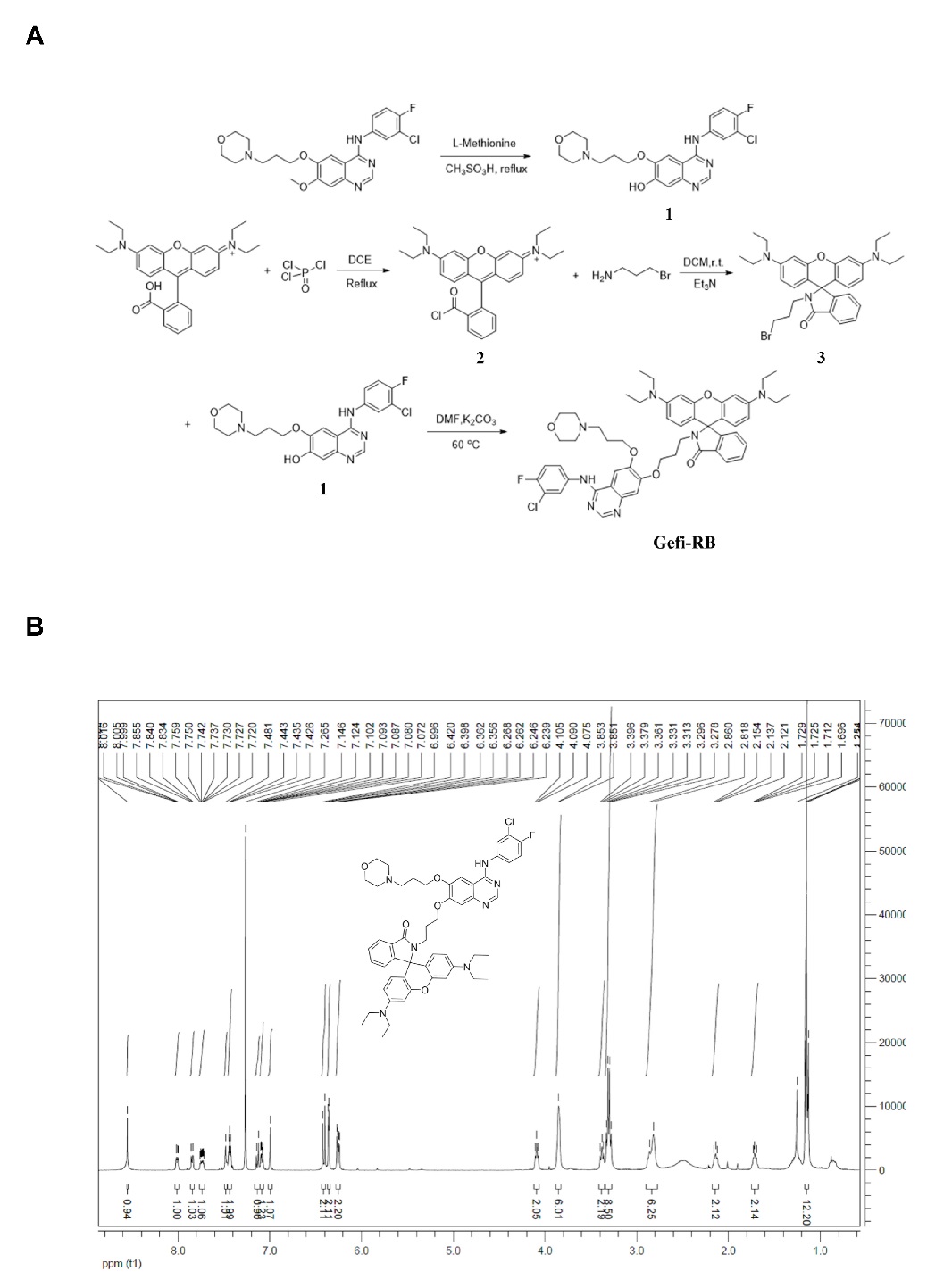
**

**Supplemental figure 6** (A) Synthesis route of compound Gefi-RB. (B) ^1^H-NMR spectra (400 MHz, DMSO-d6) of compound Gefi-RB.

**
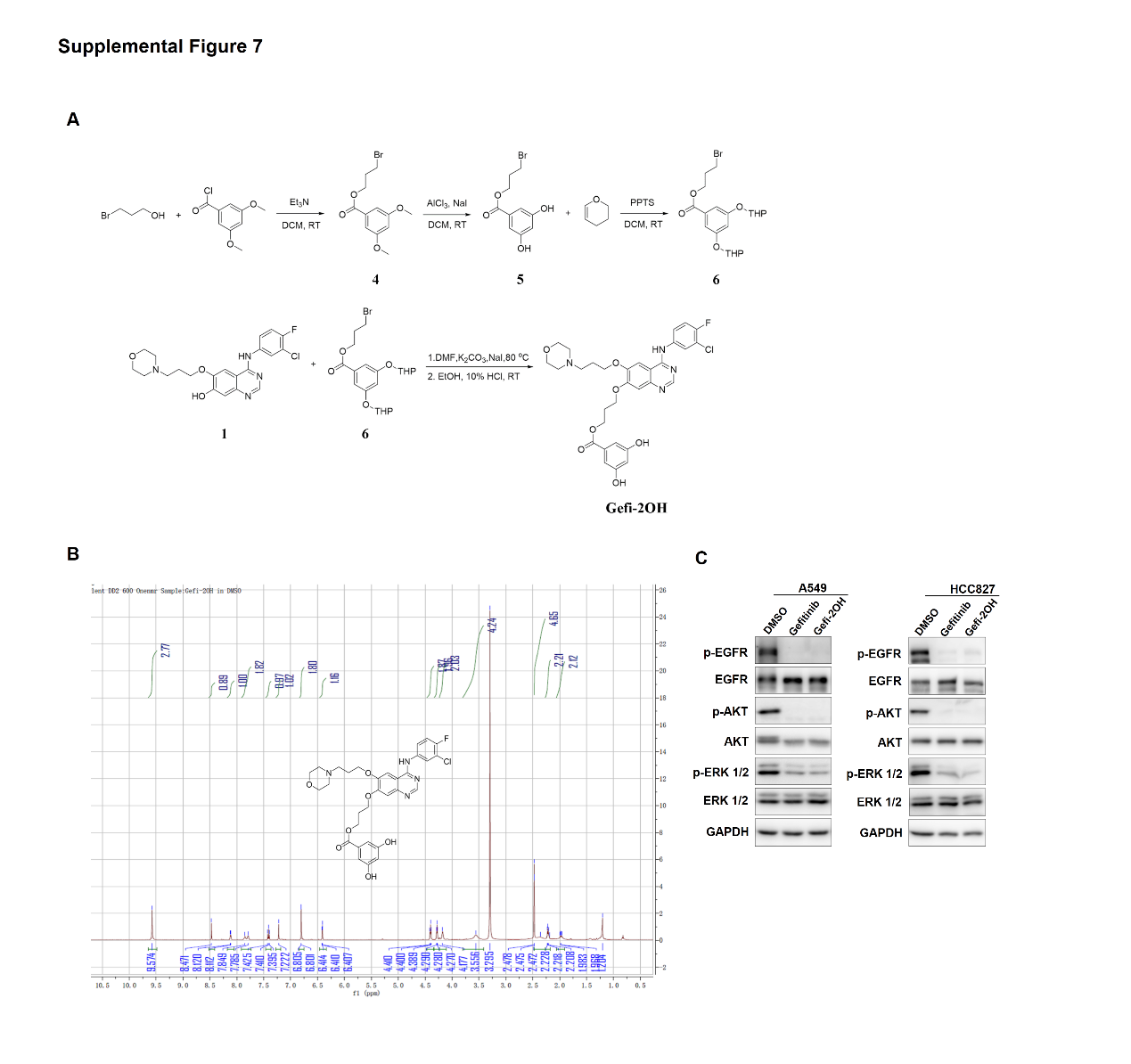
**

**Supplemental figure 7** (A) Synthesis route of compound Gefi-2OH. (B) ^1^H-NMR spectra (600 MHz, DMSO-d6) of compound Gefi-2OH. (C) A549 and HCC827 cells were treated with Gefitinib or Gefi-2OH for 24hrs and then the expression of p-AKT and the EGFR signaling pathway was measured by Western blotting.
